# Tobacco smoking, snuff dipping and the risk of cutaneous squamous cell carcinoma: a nationwide cohort study in Sweden

**DOI:** 10.1038/sj.bjc.6602475

**Published:** 2005-03-15

**Authors:** Å Odenbro, R Bellocco, P Boffetta, B Lindelöf, J Adami

**Affiliations:** 1 Department of Medical Epidemiology and Biostatistics, Karolinska Institutet, 171 77 Stockholm, Sweden; 2 International Agency for Research on Cancer, 69008 Lyon, France; 3 Department of Dermatology, Karolinska University Hospital, 171 76 Stockholm, Sweden; 4 Department of Medicine, Clinical Epidemiology Unit, Karolinska Institutet, 171 76 Stockholm, Sweden

**Keywords:** cutaneous squamous cell carcinoma, smoking tobacco, snuff dipping, epidemiology, cohort study

## Abstract

We investigated whether tobacco use causes cutaneous squamous cell carcinoma (CSCC) in a large cohort study with complete and long-term follow-up. A total of 756 incident cases occurred in a cohort of 337 311 men during a 30-year follow-up period, but no association was found between any kind of smoking tobacco use and CSCC risk, nor any risk change with increasing dose, duration or time since smoking cessation. Snuff use was associated with a decreased risk of CSCC. Overall, our study provides no evidence that tobacco use increases the risk of CSCC.

Cutaneous squamous cell carcinoma (CSCC) is a relatively common cancer worldwide, more common in males and the elderly, and a four-fold increase in age-standardised incidence has occurred in Sweden over the last four decades and this trend seems to be continuing (Cancer Incidence in Sweden, 2003). Ultraviolet (UV) radiation is recognised as the most important risk factor for CSCC (Armstrong and Kricker, 2001), although other definite or possible risk factors include immunosuppression (London *et al*, 1995), infection with oncogenic viruses (Harwood *et al*, 2000), chronic inflammatory skin diseases (Boffetta *et al*, 2001), exposure to arsenic and high body mass index (BMI) (Chen *et al* 2003).

Tobacco smoke and oral snuff contain a large number of carcinogenic substances (Wiencke, 2002; IARC, 2004). However, the relationship between tobacco use and/or oral snuff and CSCC has not been sufficiently investigated among humans and the evidence has been somewhat conflicting (Grodstein *et al*, 1995; Nilsson, 1998; Ramsay *et al*, 2000; de Hertog *et al*, 2001; Chen *et al*, 2003; Lindelöf *et al*, 2003; Struijk *et al*, 2003; IARC, 2004).

We have investigated the possible role of tobacco smoking, snuff dipping and BMI in CSCC aetiology in a large nationwide cohort of Swedish construction workers.

## SUBJECTS AND METHODS

### The cohort and follow-up

The Construction Industry's Organization for Working Environment, Safety and Health (Bygghälsan) provided outpatient health services to construction workers all over Sweden from 1969 through 1993. Beginning in 1971, the information collected was stored in a computerised register, as described elsewhere (Nyrén *et al*, 1996). The first visit to the clinic defined entry into the cohort. Since no information on smoking history was collected from 1975 to 1977, we only included the workers who were registered between 1971–1975 and 1978–1992. Since over 95% of the cohort were men, we restricted our study to male workers and overall, 337 311 men were included in the subsequent analyses. Exposure information was obtained by self-administered questionnaires double-checked by a nurse. The quality of the tobacco data has been reviewed and the answers are believed to be of high validity.

Each cohort member was followed from date of entry into the cohort until CSCC diagnosis, death, emigration or end of follow-up 31 December 2000, whichever occurred first. The unique Swedish 10-digit personal identification number was used for follow-up by record linkage to the nationwide National Death Registry (date and cause of death), the Migration Registry (date of emigration) and the Swedish Cancer Registry (Cancer Incidence in Sweden, 2003).

### Statistical analyses

The distribution of BMI was analysed in four groups according to the World Health Organization (WHO) criteria: underweight ⩽18.5; normal weight 18.6–25; overweight 25–30 and obese >30. Age was categorised in 5-year intervals. Cigarettes were assumed to contain 1 g of tobacco and cigars 6 g of tobacco each. Age-adjusted incidence rate ratios (IRR) and 95% confidence intervals (CI) were estimated using proportional hazards Cox regression model, with age and the relevant exposure factors as independent variables (*Stata Statistical Software:* Release 8.2. College Station, TX, USA: Stata Corporation). Interaction effects between tobacco variables, age and BMI were also included in the model and likelihood ratio tests were used to assess their significance.

## RESULTS

Throughout follow-up, a total of 756 men developed CSCC, the risk increasing substantially with age. The mean age at entry was 34.2 (range 14–82) years and cohort members were followed on average for 19.4 years (range 0–31.3), yielding a total of 6 536 910 person-years of follow-up. In total, 58% of the subjects had ever smoked some tobacco product and 28% were ever snuff dippers. About 43% used one tobacco product only (29% were pure cigarette smokers and 13% were pure snuff dippers) and approximately 21% mixed two or more tobacco products – most frequently cigarettes and pipe (5%) or cigarettes and snuff (12%).

Table 1 summarises the IRR and corresponding CI for all smoking tobacco taken together. Overall, no association between tobacco and the development of CSCC was found. Former and current smokers showed an IRR of 0.95 (95% CI 0.77–1.18) and 0.97 (95% CI 0.80–1.17), respectively, compared to those who never smoked. No effect could be detected with increasing dose, duration or time since cessation.

Table 2 displays the IRR and CI for the tobacco products separately and the association with BMI. Again, we found no effect of any of the different smoking tobacco products on the risk of developing CSCC. Pure cigarette smokers had an IRR of 1.04 (95% CI 0.85–1.26), pure cigar smokers 0.88 (95% CI 0.45–1.71) and pure pipe smokers 0.81 (95% CI 0.62–1.05) compared to nontobacco users. No dose–response trends were revealed.

Snuff use was found to be negatively associated with the risk of developing CSCC (IRR 0.64; 95% CI 0.44–0.95). This effect was even stronger among those who had used snuff for more than 30 years. Subjects categorised as underweight, overweight or obese were not at an increased risk of CSCC compared to those of normal weight.

## DISCUSSION

Our main finding is that the use of tobacco smoking is not associated with an increased risk of CSCC, although we found a negative association between oral moist snuff and CSCC risk. The potential impact of occupational sunlight exposure on CSCC in this cohort has been evaluated previously and no association was found (Håkansson *et al*, 2001), perhaps because outdoor workers wear protective clothing while working. Unfortunately, we do not have information on recreational sun habits among the cohort members.

Previous investigations have reported relative risks of 1.5–4.1 between cigarette smoking and CSCC (Aubry and MacGibbon, 1985; Grodstein *et al*, 1995; Ramsay *et al*, 2000; De Hertog *et al*, 2001; Struijk *et al*, 2003), whereas others found no such association (Chen *et al*, 2003; Lindelöf *et al*, 2003). In general, most studies have been case–control in design, focused only on cigarette smoking and used rather crude exposure measures; previous cohort studies were unable to study skin cancer since they mainly used mortality instead of incidence data.

In the few studies of pipe and cigar smoking and BMI as risk factors of CSCC (De Hertog *et al*, 2001; Efird *et al*, 2002; Chen *et al*, 2003), pipe smoking and underweight have been associated with elevated risk of CSCC in contrast to our results. No studies have specifically addressed the impact of snuff use on CSCC.

The major strengths of this study of Swedish construction workers are its large size, the prospective collection of exposure details and almost complete follow-up. Any residual misclassification would presumably be nondifferential and would not have any major impact on the findings.

## Figures and Tables

**Table 1:** Estimated age-adjusted incidence rate ratios (IRR) and corresponding 95% confidence intervals (CI) for developing CSCC, by different dose and duration of tobacco smoking

**Exposure variable**	**No of cases (%)**	**Person-years accumulated**	**IRR (95% CI)**
*Reference (never tobacco user)*	209 (35)	1 920 810	1 (ref)

*Any tobacco smoker*			
Previous	141 (24)	710 190	0.95 (0.77–1.18)
Current	245 (41)	2 150 910	0.97 (0.80–1.17)

*Total smoking tobacco dose (g day* ^−*1*^ *)*			
⩽10	222 (39)	1 410 290	0.99 (0.82–1.19)
11–15	112 (20)	1 119 380	0.88 (0.70–1.11)
>15	27 (5)	230 650	0.95 (0.64–1.42)

*Years of tobacco smoking*			
⩽15	87 (15)	1 534 570	1.08 (0.83–1.39)
16–25	110 (19)	725 130	1.11 (0.87–1.40)
>25	188 (32)	563 890	0.87 (0.72–1.07)

*Time since cessation of tobacco smoking (years)* a			
<10	74 (21)	487 210	1.00 (0.76–1.30)
⩾10	66 (19)	197 480	0.89 (0.68–1.18)

a Stratified Cox proportional hazards model, by age.

CSCC=cutaneous squamous cell carcinoma.

**Table 2:** Estimated age-adjusted incidence rate ratios (IRR) and 95% confidence intervals (CI) for developing CSCC by different types of tobacco

**Tobacco variable**	**No. of cases^a^**	**Person-years accumulated**	**IRR (95% CI)**
*Reference (nontobacco users)*	209	1 920 810	1 (ref)
*Cigarette smoker*	194	1 947 400	1.04 (0.85–1.26)^b^

*Cigarettes smoked per day* b ^,^ c			
<10	105	836 590	1.12 (0.88–1.41)
11–20	58	667 330	0.92 (0.69–1.24)
>20	26	405 710	0.90 (0.60–1.36)

*Cigar smoker*	9	42 000	0.88 (0.45–1.71)^b^

*Pipe smoker*	80	358 200	0.81 (0.62–1.05)^b^

*Pipe tobacco (g) smoked per week*			
<80	75	338 180	0.77 (0.59–1.00)
⩾80	5	19 790	1.12 (0.46–2.73)

*Snuff dipper*	29	661 150	0.64 (0.44–0.95)^b^

*Years of snuff dipping*			
<30	14	610 320	0.79 (0.46–1.38)
⩾30	15	44 660	0.58 (0.34–0.99)

*Mixed user*	235	1 607 340	1.08 (0.90–1.30)^b^

*BMI*			
⩽18.5	3	93 390	0.87 (0.28–2.71)
18.6–25	372	4 153 490	1-ref
26–29	326	1 986 410	0.95 (0.81–1.10)
>30	52	282 310	0.98 (0.73–1.32)

a The discrepancy in number of cases is due to missing information on dose.

b Adjusted for all other tobacco categories

c Stratified Cox proportional hazards model, on age.

BMI=body mass index; CSCC=cutaneous squamous cell carcinoma.
